# Evolution Mechanism of Arsenic Enrichment in Groundwater and Associated Health Risks in Southern Punjab, Pakistan

**DOI:** 10.3390/ijerph192013325

**Published:** 2022-10-15

**Authors:** Muhammad Yousuf Jat Baloch, Wenjing Zhang, Dayi Zhang, Baig Abdullah Al Shoumik, Javed Iqbal, Shuxin Li, Juanfen Chai, Muhammad Ansar Farooq, Anand Parkash

**Affiliations:** 1 Key Laboratory of Groundwater Resources and Environment, Ministry of Education, Jilin University, Changchun 130021, China; 2 College of New Energy and Environment, Jilin University, Changchun 130021, China; 3 Soil, Water and Environment Discipline, Khulna University, Khulna 9208, Bangladesh; 4 School of Environmental Studies, China University of Geosciences, Wuhan 430074, China; 5 Institute of Environmental Sciences and Engineering, School of Civil and Environmental Engineering, National University of Science and Technology, Islamabad 44000, Pakistan; 6 School of Chemistry and Chemical Engineering, Shaanxi Normal University, Chang’an West Street 620, Xi’an 710119, China

**Keywords:** arsenic contamination, hydrogeochemistry, geochemical modelling, inverse modelling, health risk assessment

## Abstract

Arsenic (As) contamination in groundwater is a worldwide concern for drinking water safety. Environmental changes and anthropogenic activities are making groundwater vulnerable in Pakistan, especially in Southern Punjab. This study explores the distribution, hydrogeochemical behavior, and pathways of As enrichment in groundwater and discusses the corresponding evolution mechanism, mobilization capability, and health risks. In total, 510 groundwater samples were collected from three tehsils in the Punjab province of Pakistan to analyze As and other physiochemical parameters. Arsenic concentration averaged 14.0 μg/L in Vehari, 11.0 μg/L in Burewala, and 13.0 μg/L in Mailsi. Piper-plots indicated the dominance of Na^+^, SO_4_^2−^, Ca^2+^, and Mg^2+^ ions in the groundwater and the geochemical modeling showed negative saturation indices with calcium carbonate and salt minerals, including aragonite (CaCO_3_), calcite (CaCO_3_), dolomite (CaMg(CO_3_)_2_), and halite (NaCl). The dissolution process hinted at their potential roles in As mobilization in groundwater. These results were further validated with an inverse model of the dissolution of calcium-bearing mineral, and the exchange of cations between Ca^2+^ and Na^+^ in the studied area. Risk assessment suggested potential carcinogenic risks (CR > 10^−4^) for both children and adults, whereas children had a significant non-carcinogenic risk hazard quotient (HQ > 1). Accordingly, children had higher overall health risks than adults. Groundwater in Vehari and Mailsi was at higher risk than in Burewala. Our findings provide important and baseline information for groundwater As assessment at a provincial level, which is essential for initiating As health risk reduction. The current study also recommends efficient management strategies for As-contaminated groundwater.

## 1. Introduction

Water is a critical resource for human life, but chemical and microbial contamination frequently challenges its safety [1,2,3,4]. Specifically, groundwater is one of the most commonly used water resources for drinking [5,6,7]. Therefore, there is always a need to provide and supply clean groundwater for drinking [8,9]. Arsenic (As) is a naturally occurring element with high toxicity and carcinogenic potential [10,11]. Groundwater As pollution has been a severe worldwide health and environmental problem for many years [12,13]. Drinking As-contaminated water has been linked to various health problems in Asian countries [14]. Chronic exposure to As through drinking groundwater may lead to health risks, such as kidney cancer, skin lesions, neurological diseases, and liver damage, and death may occur in the most severe cases [15]. As release in aquifers is caused by geochemical changes in underground sediments and is usually ascribed to the process of the reductive dissolution of arsenic-bearing minerals in aquifers [16,17,18]. A few examples include the water–rock interactions, sorbent/desorbent processes, residence time along the flow channel, oxidative/reductive dissolution processes, and the variation in the chemical composition of groundwater containing arsenopyrite (FeAsS), and calcium carbonate (CaCO_3_^−^), all of which can release As in groundwater systems [19,20,21]. As also enters into aquifers from human activities like mining and smelting, industrial waste disposal, fertilizer use, agricultural operations, and wastewater irrigation [18]. As in the aquatic environment is mainly found in the forms of arsenite (As(III)) and arsenate (As(V)). As(III) is more widespread in reduced environments, whereas As(V) is abundant in oxidized environments [22,23].

As-contaminated groundwater threatens millions of people’s health and lives, especially in densely populated places. Over 200 million people in 105 countries are affected, and, in Asia, the health of about 150 million people is at risk through the consumption of As-contaminated groundwater [24,25,26]. Although some countries have lower arsenic concentrations in the groundwater (<10 μg/L), many others, including Bangladesh [27], China [28], Egypt [29], India [30], Indonesia [31], Nepal [32], and the Philippines [33] have over than 50 μg/L of As in groundwater aquifers. Pakistan is also one of the As-contaminated countries, facing water scarcity and groundwater vulnerabilities [34]. A recent study on groundwater pollution in Pakistan emphasized the problem of As contamination, especially in the Punjab and Sindh provinces connected to the Indus River and its tributaries [35,36,37]. In these contaminated regions, arsenic concentration was estimated 2580 μg/L in Lahore and Kasur (Punjab) [38,39], 905 μg/L in Muzaffargarh (Punjab) [40], 201 μg/L in Chichawatni (Punjab) and 29.30 μg/L in Dera Ghazi Khan (Punjab) [35,41]. In addition, arsenic concentration was as high as 1516 μg/L in Jamshoro (Sindh) [42], 158 μg/L in Manchar lake (Sindh) and 2580 μg/L in Tharparkar (Sindh) [43,44]. In Hyderabad, over 40 people died in 2004 because of polluted drinking water with high levels of As [45]. About 70% of Pakistan’s groundwater and surface water is contaminated with biological, organic and inorganic contaminants [46]. According to the current state of As contamination in Pakistan’s drinking water, about 47 million people live in places where more than 50% of well water contains >10 μg/L of As [46] and 17% surpasses 50 μg/L [47]. Only 26% of Pakistan’s population has access to safe drinking water [48]. The health risk map for As concentration in 1187 instances of groundwater suggests that the Indus Basin is severely contaminated by As [49]. The elevated As concentration may be attributable to oxidative desorption driven by many physicochemical parameters [47]. Numerous reports have explored As contamination in groundwater in various places, including rural and urban areas [24], peri-urban areas [50], rivers [47] and health facilities [51], while some earlier studies established As distribution on a small scale without comparisons between tehsils in Punjab’s groundwater and health risk assessments.

Considering the above-mentioned circumstances, this study explored As distribution, formation and evolution mechanisms in groundwater through geochemical modeling and multivariate analysis. We aimed to unravel the As contamination situation in Punjab’s groundwater and conduct an assessment on the health risks caused by the consumption of As-contaminated groundwater.

## 2. Materials and Methods

### 2.1. Study Area

**Figure 1:**
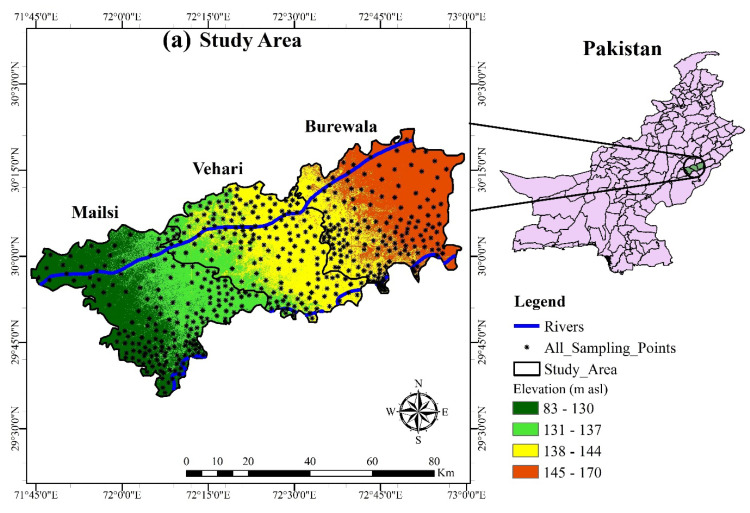

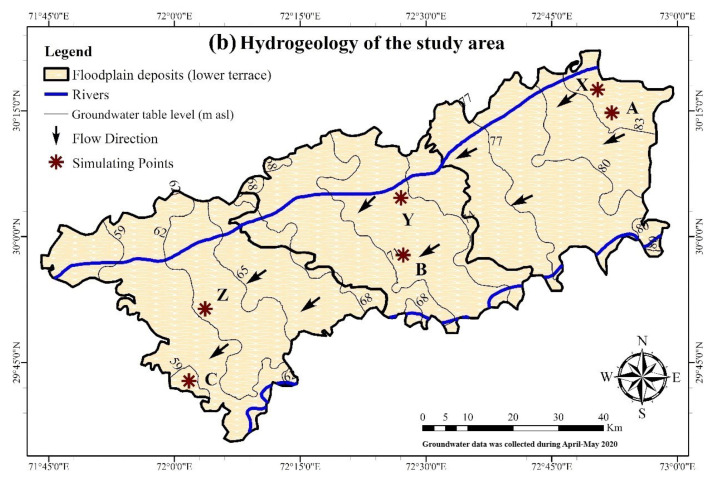
(**a**) Groundwater sampling points of the study area. (**b**) Flow and hydrogeology map.

Vehari, Burewala and Mailsi are three sub-districts (tehsils) of the Punjab Province in Pakistan. The area is bounded by the Sutlej and Ravi rivers and is located at 83–170 m asl (altitude) between 30°04′19″ N and 72°35′28″ E, as illustrated in Figure 1. The total population is 3 million and the climate is hot in the summer, from April to August, rising to a maximum of 50 °C, and cold in winter, from November to February, dropping to a minimum of about 5 °C. Dust storms are typical throughout the summer, and the annual average rainfall is around 125 mm, most of which falls during the monsoon season (July and August) [52]. Electric pumps and tube wells are used to exploit groundwater.

The Indus and Jhelum rivers pass around the study area. In contrast, the Ravi and Satluj rivers run through the study area, the rainwater and rivers serves as recharge sources for the aquifer. The south Indus River forms the plain alluvial deposition and its five major tributaries, which contain sediments from the Pleistocene and Holocene, are carried by the Ravi and Sutlej rivers. Unconsolidated alluvial deposits form the aquifer with varying amounts of sand with a large proportion of fine sand and silt and low organic matter. Since the late tertiary period, these deposits have been formed through the Indus River and its tributaries in the wide alluvial plain, which stretches from the Himalayan foothills to the Arabian Sea. The minerals, including anhydrite, aragonite, calcite, dolomite, goethite, gypsum, halite, and hematite, were detected in the mineralogical assessment [24,53]. The tributaries of the Indus River deposited a 400-m thick layer of sediment throughout the Pleistocene. Groundwater originated in interbedded silt and alluvial sand layers that are well-distributed across the Punjab area. This region’s residents depend on groundwater for drinking, livestock, and agriculture.

### 2.2. Groundwater Samples

In total, 510 groundwater samples were collected from shallow aquifers (<35 m) of three tehsils, namely Vehari, Burewala, and Mailsi from April–May 2020 and filtered to 0.45 μm for further analysis. A global positioning system (GPS) was used to record the groundwater sample’s location. (APHA et al. 2005) The American Public Health Association’s standard methods were followed [54]; 250 and 100 mL glass bottles were used to collect samples for the analysis of cations and anions, respectively. A drop of nitric acid (HNO_3_) was immediately added to the samples to adjust pH (<2.0) for cation analysis.

### 2.3. Hydrogeochemical Processing and Health Risk Assessment 

All analyses followed the methods prescribed by the APHA 2005 [54]. The samples were analyzed at the Pakistan Council of Research in Water Resources (PCRWR) Islamabad, Pakistan. The calibrated portable meters (RS232C/Meter CON 110) were used to measure water pH, turbidity, electric conductivity and total dissolved solids on-site. Arsenic and major cations (Na^+^, Ca^2+^, Mg^2+^, K^+^, Fe^2+^) were analyzed by inductively coupled plasma atomic emission spectrometry (ICP-AES/27). An ion chromatograph (IC/P60) was used to measure SO_4_^2−^, Cl^−^, NO^3−^, and F^−^, while HCO_3_^−^ and total alkalinity were measured using the acid-base titration method. Finally, the samples were further examined for accuracy by computing percentage charge balance errors (CBE), as seen in Equation (1).

(1) $$\%\mathrm{CBE}=\frac{\left[\sum \mathrm{cations}-\sum \mathrm{anions}\right]}{\left[\sum \mathrm{cations}+\sum \mathrm{anions}\right]\;}\times 100$$

Here, the unit of cations and anions is meq/L and the physicochemical analysis with a %CBE within ±5% are considered perfect for further research.

SPSS v16.0 (SPSS Inc., Chicago, IL, USA) was used for the statistical analysis of groundwater physicochemical parameters. XLSTAT v19 was used for Pearson’s correlation and principal component analysis (PCA) to determine the factors influencing groundwater quality. Piper, 1994 [55], was incorporated, using Golden Software Grapher 18.3 (Golden, CO, USA). Origin v19 (Sydney, Australia) plotted the ionic ratios to describe the dominant ionic water facies displaying the water chemistry in the study area. 

According to USEPA (2005), the chronic daily intake (CDI), hazard quotients (HQ), and cancer risk (CR) were evaluated [56,57]. Two models were used for the health risk assessment of exposure to uncover As-contaminated groundwater. Carcinogenic and non-carcinogenic health hazards were determined by CDI, as described in Equation (2). Non-carcinogenic risk was estimated by HQ, as listed in Equation (3), and carcinogenic risk was calculated by CR, as described in Equation (4).

(2) $$\mathrm{CDI}=\frac{C\;\times \mathrm{IR}}{\mathrm{BW}}$$

(3) $$\mathrm{HQ}=\frac{\mathrm{CDI}}{\mathrm{RfD}}$$

(4) CR=CDI×CSF

Here, C represents the As concentration in groundwater (mg/L), and IR represents daily water consumption, 0.5 L/day for children and 2 L/day for adults. BW represents body weight, 20 kg for children and 70 kg for adults. RfD refers to the oral reference dose of As, 0.3 mg/kg/day for children and adults, and CSF denotes the cancer slope factor for As, 1.5 mg/kg/day for children or adults [57]. Groundwater samples with (HQ < 1.0) were considered safe to drink, and groundwater samples with (CR > 10^−4^) exhibited considerable cancer risk [57]. 

### 2.4. Geochemical Modeling 

PHREEQC version 3.0 software package was used to calculate the mineral saturation level [58]. The saturation index was used to illustrate the representative mineral phases, including anhydrite, aragonite, calcite, dolomite, goethite, gypsum, halite, and hematite, which are essential characteristics in the study area. The saturation indices (SI) that explain the thermodynamic tendency of minerals to precipitate or dissolve were calculated using Equation (5).

(5) $$\mathrm{SI}=\mathrm{Log}\frac{\mathrm{IAP}}{\;\mathrm{Ksp}\;}=\mathrm{LogIAP}-{\mathrm{LogK}}_{\mathrm{Sp}}$$

Here, IAP represents the ion activity product for the solution of dissociated types, and Ksp represents the equilibrium solubility product at groundwater sample temperature for the chemicals involved. The hydrochemical equilibrium state was at SI = 0, whereas negative (SI < 0) and positive (SI > 0) values indicated the under-saturation and oversaturation of the minerals, respectively.

The inverse groundwater geochemical evolution modeling over two flow pathways along the (A to C) and (X to Z) were carried by PHREEQC (Figure 1). According to the groundwater flow pattern and hydrogeological conditions, the selected path I flows from A to B and B to C, and the selected path II flows from X to Y and Y to Z. Major ions (Na^+^, Mg^2+^, Ca^2+^, Fe^2+^, K^+^, HCO_3_^−^, SO_4_^2–^, Cl^−^, and NO_3_^−^) were used in the model simulation. The mineral phases include anhydrite, aragonite, calcite, dolomite, goethite, gypsum, halite, and hematite. Due to the wide-ranging distribution of the clay minerals in the study area, the exchange of cation is also considered. For significant tendencies, our model moreover considers the CaX_2_/NaX, CO_2_(g), and H_2_O(g) phases [59].

## 3. Results and Discussion 

### 3.1. Characteristics of Groundwater Geochemistry

Groundwater hydrogeochemical properties in three tehsils are listed in Table 1 and compared to the WHO 2022 recommended drinking water quality standard [60]. EC ranged from 308 to 4550 μS/cm (averaging 1570 μS/cm), 85 to 4400 μS/cm (averaging 1231 μS/cm), and 226 to 2690 μS/cm (averaging 1185 μS/cm) in Vehari, Burewala, and Mailsi, respectively, exceeding recommended guidelines. Such huge changes in EC are usually caused by human activity and mineral dissolution in groundwater. Our findings imply that geochemical reactions, rock–water interactions, and anthropogenic sources influence the groundwater chemistry in the study area [61]. However, higher TDS in Vehari ranged from 234 to 3148 mg/L (averaging 1072 mg/L), Burewala 277 to 3173 mg/L (averaging 896.68 mg/L), and Mailsi 359 to 3298 mg/L, (averaging 896 mg/L). Water pH values in Vehari, Burewala, and Mailsi ranged from 6.85 to 7.61 (averaging 7.17), 6.78 to 7.11 (averaging 6.98), and 7.2 to 8.35 (averaging 7.66), respectively. The chemical composition of groundwater is pH-dependent, the groundwater chemistry fluctuates with changes in pH, and this variance is mostly driven by different chemicals, such as fertilizer for agriculture activities [5]. In the study area, the groundwater pH was alkaline, which might be caused by the presence of HCO_3_^−^ and rock weathering. The turbidity in Vehari (26.37 NTU) and Burewala (15 NTU) were higher, whereas the turbidity in Mailsi (1.22 NTU) was lower than the WHO guidelines. Elevated EC and TDS levels in groundwater are caused by electrolytes and high salinity, which are often associated with semi-arid and dry climatic conditions and are responsible for the increased turbidity in Vehari and Burewala. Furthermore, the alkaline environment may increase conductivity over time by hastening the dissolving process. The obtained higher EC values here might be explained by the presence of more dissolved salts in groundwater. High TDS levels are most likely caused by wastewater discharged into pits from residential and dyeing units, ponds, and lagoons. The average value of total hardness in Vehari, Burewala, and Mailsi was 368.47, 348.85, and 414.58 mg/L, respectively. The higher concentration of bicarbonate in the groundwater may be attributed to rock weathering, atmospheric sources, and anthropogenic activities. The average HCO_3_^−^ contents were: Vehari, 345.08 mg/L; Burewala, 299.20 mg/L; and Mailsi (324.23 mg/L). The majority of HCO_3_^−^ comes from geological sources, such as carbonate dissolution and carbonate cement formations. Both anion and cations observed consistent sequences—cations: Na^+^ > Ca^2+^ > Mg^2+^ > K^+^ > Fe^2+^; and anions: HCO_3_^−^ > SO_4_^2−^ > Cl^−^ > NO_3_^−^ > F^−^. As concentrations in Vehari, Burewala, and Mailsi ranged to 45 μg/L (averaging 14.0 μg/L), 52 μg/L (averaging 11.0 μg/L), and 89 μg/L (averaging 13.0 μg/L), respectively. They all exceeded the permitted limit of 10 μg/L. More precisely, As concentrations in Vehari and Mailsi was significantly higher than Burewala because of high bicarbonate (HCO_3_^−^) content and pH [62]. In this study, the high concentrations of HCO_3_^−^ and alkaline water quality indicated an oxidative condition in aquifers. Most areas in Southern Punjab belong to tributary areas, where groundwater has high minerals and As concentrations, which are not suitable for drinking. Thus, Vehari and Mailsi face the critical challenge of the most widespread As contamination. Although a prior investigation indicated a significant Fe^2+^ content in Jamshoro [42] and compared to other As-affected places the southeast Asia region [63], Fe^2+^ iron in our study was lower than that report, while the high HCO_3_^−^ may dissolve the carbonate minerals, possibly explaining the higher As concentration in groundwater.

### 3.2. Hydrogeochemical Origin of Groundwater 

#### 3.2.1. Geochemical Classifications of Groundwater

**Figure 2:**
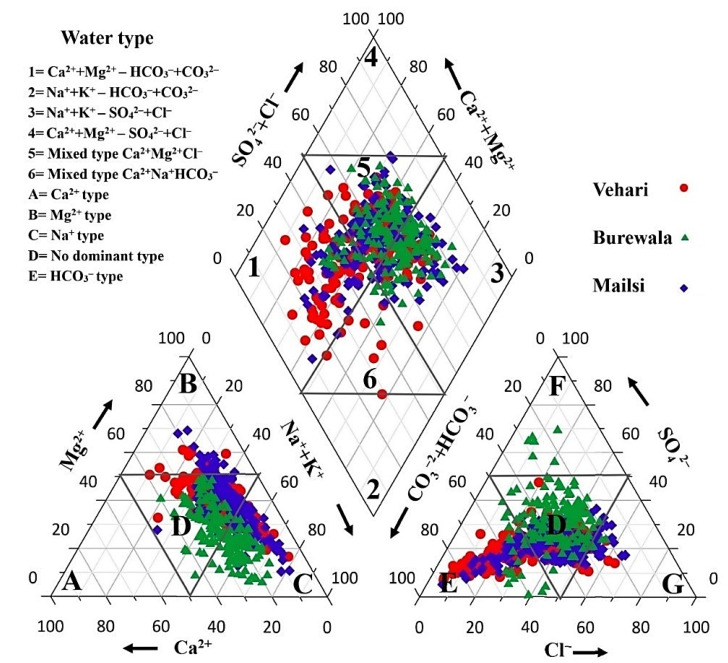
Piper diagram showing groundwater chemistry in study area.

Piper (1994) proposed a convenient method to classify and compare water chemistry types based on an ionic composition by plotting the hydrogeochemical data on a trilinear diagram [55]. The first type of the triangle in Piper’s plot represents anions and the second type represents cations (Figure 2). The groundwater type is dominated by Na^+^, Ca^2+^, and Mg^2+^ amongst cations, while HCO_3_^−^, SO_4_^2−^, and Cl^−^ amongst anions. Cation concentrations were in the order of Na^+^ > Ca^2+^ >Mg^2+^ > K^+^ > Fe^2+^, and the anions were ranked as HCO_3_^−^ > SO_4_^2−^ > Cl^−^ > NO^3−^ > F^−^. Rasool et al. reported a similar pattern of anion and cation concentrations [64]. In addition, groundwater showed mixing behavior to Ca^2+^, SO_4_^2−^, and Mg^2+^, which explained the geochemical formation with calcium, magnesium, and bicarbonate-containing mineral phases [65]. The Na^+^ and Cl^−^ type of groundwater indicated that rock–water interaction and agricultural activities played significant roles in the study area. In Piper’s plot, Na^+^−SO_4_^2−^ and Ca^2+^ and Mg^2+^ types of waters indicated As release by the sedimentary rocks into groundwater. Several important mechanisms result in As mobilization in groundwater, such as the dissolution of calcium, the dissolution of salt minerals, and desorption due to high pH [66,67]. 

#### 3.2.2. Ionic Ratios and Mineral Phases of Groundwater

**Figure 3:**
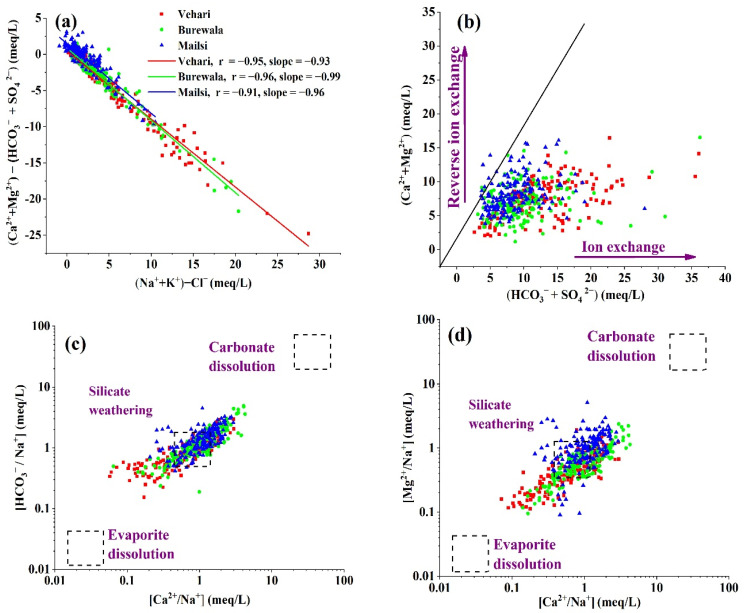
Scattered ionic plots. (**a**) [(Ca^2+^ + Mg^2+^) − (HCO_3_^−^ + SO_4_^2^^−^)] vs. [(Na^+^ + K)−Cl^−^]. (**b**) [(Ca^2+^ + Mg^2+^ vs. HCO_3_^−^ + SO_4_^2^^−^)]. (**c**) [(HCO_3_^−^/Na^+^) vs. (Ca^2+^/Na^+^)]. (**d**) [(Mg^2+^/Na^+^) vs. (Ca^2+^/Na^+^)].

The main factors determining arsenic concentration in the groundwater of the study area were identified using ionic ratios. The plots [(Ca^2 +^ + Mg^2+^) − (HCO_3_^−^ + SO_4_)] and [(Na + K) − Cl] were employed to identify the exchange of cations in aquifers. [(Ca^2+^ +Mg^2+^) − (HCO_3_^−^ + SO_4_)] illustrated that in the system, Ca^2+^ and Mg^2+^ amounts were removed/added separately from calcite, dolomite, and gypsum, whereas [(Na + K) − Cl^−^] illustrated the sodium removed/added in the system separately to the chloride salts [68]. The ionic exchange of Na^+^, Ca^2+^, and Mg^2+^ within the system was indicated by the linear slope of −1. Therefore, our findings suggested the strong effect of cation exchange on significant correlations in Vehari (−0.95, slope = −0.93), Burewala (−0.96, slope = −0.99), and Maisli (−0.91, slope = −0.96) (Figure 3a). The importance of cation exchanges was further emphasized by the (Ca^2+^ + Mg^2+^ vs. SO_4_ + HCO_3_^−^) plot (Figure 3b). Most groundwater samples were below the line of 1:1, showing the effects of silicate weathering and cation exchange in the study area [68]. The carbonate dissolution is the main supplier of sodium, magnesium, calcium, and bicarbonate ions into the groundwater. The plots revealed that silicate weathering for the contamination of the groundwater was influenced by dissolved carbonate minerals (HCO_3_^−^/Na^+^) vs. (Ca^2+^/Na^+^) (Figure 3c) and (Mg^2+^/Na^+^) vs. (Ca^2+^/Na^+^) (Figure 3d).

**Figure 4:**
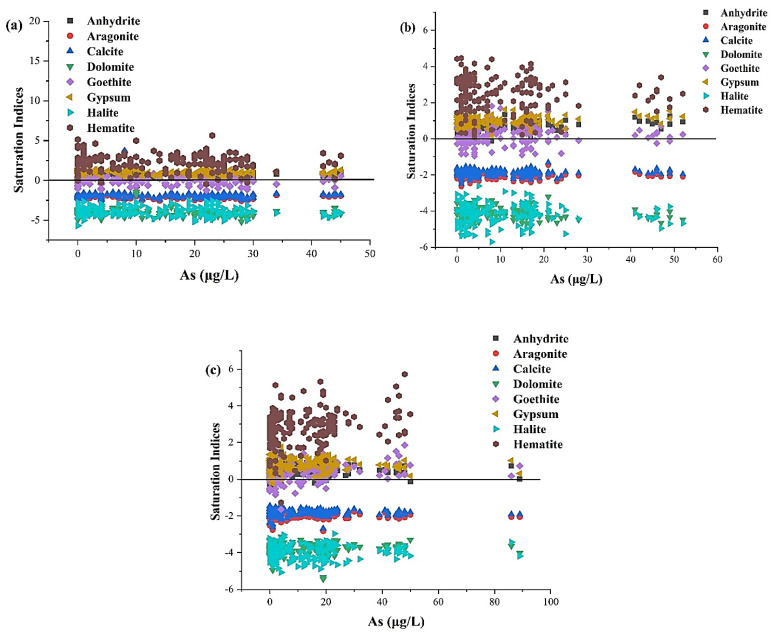
Relationships between As and saturation indices in groundwater. (**a**) Vehari, (**b**) Burewala, and (**c**) Mailsi.

The groundwater hydrogeochemistry was also supported by the saturation index (SI) and always affected by the minerals [69]. The SI estimation facilitates understanding the reaction pathways and the measurement of mineral dissolution and precipitation. In the geochemical simulation model (Table S1), aquifer conditions were undersaturated (SI < 0) with calcium carbonate and rock salt minerals, including aragonite (CaCO_3_), calcite (CaCO_3_), dolomite ((CaMg(CO_3_)_2_), and halite (NaCl). These mineral phases had negative SI values and were unlikely to precipitate, possibly playing a vital role in releasing As into aquifers due to their dissolution [70,71]. In contrast, the SI was positive for anhydrite (CaSO_4_), gypsum (CaSO_4_2H_2_O), and iron oxide mineral phases, including goethite (FeOOH) and hematite (Fe_2_O_3_). These minerals tended to participate in groundwater (Figure 4a–c). Hasan et al. (2009) found that iron oxides in the sediments of the flood plain in Bangladesh inhibited As mobility in groundwater [72]. 

#### 3.2.3. Correlation Analysis

In Pearson’s analysis, correlation was marked as weak (r < 0.3), moderate (r **≥** 0.5), and strong (r ≥ 0.7) [73]. From the correlation matrices for Vehari, Burewala, and Mailsi (Tables S2–S4), we were able to understand the geochemical process in the study area. In Vehari, As exhibited a negative correlation with EC, HCO_3_^−^, SO_4_^2^^−^, Ca^2+^, and Fe^2+^ (*p* < 0.05), TDS, Mg^2+^, K^+^, and hardness (*p* < 0.01) (Table S2). Such correlations highlighted the influence of pH on As concentration in groundwater, matching well with a previous study [49]. In contrast, groundwater As in Burewala exhibited significant positive correlations with Ca^2+^ and hardness (*p* < 0.01) but negative correlations with NO_3_^−^ and F^−^ (*p* < 0.05) (Table S3). In Mailsi, As was positively correlated with turbidity (*p* < 0.01) and Mg^2+^ (*p* < 0.05) but negatively correlated with Ca^2+^ (*p* < 0.05) and NO_3_^−^ (*p* < 0.05) (Table S4). In all three tehsils, EC had a significant positive correlation with TDS, cations, and anions at the 1% level but not with Fe^2+^. The correlation of EC with other parameters indicated the higher possibilities of ion exchange in the aquifers [74]. 

#### 3.2.4. Principle Component Analyses (PCA)

To identify the probable As sources in aquifers, PCA was used as a multivariate method lowering the number of independent variables to limited fundamental components [73]. As listed in Table 2, the three main components (PC1 to PC3) explained 59.15% of the variance in groundwater in Vehari. PC1 explained 38.04% of the variance and mainly consisted of EC (0.964), TDS (0.981), SO_4_^2−^ (0.936), Cl^−^ (0.867), Na^+^ (0.856), Mg^2+^ (0.816), HCO_3_^−^ (0.707), and hardness (0.662), indicated the process of As geogenic in groundwater. PC2 contributed to 12.83% of total variance, mainly consisted of Ca^2+^ (0.831) and hardness (0.71). Thus, calcium was closely linked with hardness and mostly originated in sedimentary rocks. PC3 explained 8.2% of the total variance, predominated by As (0.514), pH (0.376), and turbidity (0.329). It hinted at geogenic origin and enrichment of As in Vehari.

In Burewala, the first three components explained 63.15% of the total variance. PC1 contributed 44.49% of the total variance, mainly comprising EC (0.957), TDS (0.962), alkalinity (0.837), HCO_3_^−^ (0.821), Cl^−^ (0.844), SO_4_^2–^ (0.856), Ca^2+^ (0.592), Mg^2+^ (0.833), and Na^+^ (0.859). This indicated that mineral weathering and the rock–water interaction determine cation and anion distribution. PC2 was 10.88% of the total variance, including Ca^2+^ (0.706), hardness (0.527), and As (0.527), which explained As release by the sedimentary rocks in aquifers. PC3 explained 7.77% of the total variance and consisted of NO_3_^−^ (0.648). This might be explained by the inappropriate usage of nitrate fertilizers, such as urea for irrigation purposes and anthropogenic activities, hinting at their significant impacts on groundwater quality [48]. 

Among the three main components explaining 62.86% of total variance in Mailsi, PC1 contributed 44.51% of the total variance and consisted of EC (0.869) and TDS (0.972). This results met well with the positive links between EC, TDS and hardness, evidencing the roles of water–rock interactions in substantial HCO_3_^−^ due to the dissolution of carbonate minerals [39]. PC2 explained 11.33% of the total variance and included turbidity (0.745) and As (0.783), indicating that arsenic was mobilized geologically [42]. PC3 explained 7.01% of the total variance and consisted of pH (0.512) and Mg^2+^ (–0.502).

Taking all results in the three regions together, EC and TDS were positively linked with both cations and anions in the groundwater. Previous studies have used PCA and the hierarchical cluster analysis (HCA) to identify potential As sources in the groundwater. Bibi et al. (2015) suggested that As in groundwater was geogenic because of the weathering of sulphide minerals [75]. Brahman et al. (2013) also used PCA to investigate and report high concentrations of SO_4_^2–^ (398–409 mg/L), low Fe^2+^ concentration (0.097–0.132 mg/L), and high pH values (7.9–9.2) in the groundwater of Tharparkar (Sindh) [44]. These conditions indicate that As might be released in the oxidizing conditions of the quaternary sediment [76,77]. In this study, we observed similar levels of EC and TDS, anions, and cations in groundwater, hinting at the geogenic sources of As release in groundwater.

#### 3.2.5. Groundwater and Arsenic Evolution along Flow Path

PHREEQC was used for geochemical inverse modeling [58], and two predefined flow lines were introduced in model to understand the effects of hydrogeochemical evolution on As mobilization along groundwater flow paths in this study. Six groundwater samples were used for geochemical inverse modeling (A-B-C and X-Y-Z) based on flow paths I and II, respectively. Briefly, four hydrogeochemical processes included: (1) the dissolution of calcium and salt bearing-minerals, (2) chemical weathering, (3) exchange of cations between Ca^2+^ and Na^+^, and (4) CO_2_ accounting for the interaction of atmosphere or microbial respiration resulting from organic decomposition. Modeling results (Figure 1 and Table S5) illustrated that anhydrite, gypsum, and iron oxide minerals precipitated, while other rock salt minerals, including goethite, hematite, halite, and NaX, remained in the recharge zone. Cation exchange was also involved in this process, Na^+^ (0.03124 mmol/L) was dissolved in groundwater being released from the soil and rock surface through the dominance of Ca^2+^, Na^+^, K^+^, and HCO_3_^−^ flow of groundwater. When groundwater moved from B to C, where anhydrite, goethite, and hematite precipitated, the cation ion exchange Ca^2+^ and Na^+^ were released into groundwater. Generally, the increase of As from A to C was attributed to the dissolution process of calcium-bearing minerals.

In Path II X–Y, anhydrite precipitation and the cation exchange CaX_2_ (0.01642 mmol/L) was specified by the Na^+^ + K^+^-HCO_3_ facies. As the groundwater moved from Y to Z, the carbonate minerals were dissolved through the stronger exchange of cations, Ca^2+^ interchanged with Na^+^. The As concentration in groundwater was elevated along with the flow path X–Z, as evidenced by the increased HCO_3_^−^, Cl^−^, and TDS. This result suggested that the additional sources of As were introduced into groundwater [59]. The increasing HCO_3_^−^, Cl^−^, and TDS concentrations in groundwater might be related to the dissolution of minerals [70,71]. Notably, the dissolving of calcium carbonate and salt mineral (halite) is evident from the groundwater conditions. Our results are well supported by our saturation indices (SI < 0) results, which indicated the dissolution of calcium carbonate and rock salt minerals, possibly playing a vital role in releasing As into groundwater. This implies that the groundwater condition is affected by the higher dissolution of calcium carbonate and salt minerals regarding the As release into groundwater. 

### 3.3. Mechanism of As Enrichment and Geospatial Distribution

Iron concentration is low in groundwater when groundwater oxygenation is available [78]. In this study, rivers pass through the study area, and the clay-covered aquifer top layer allows aerobic activities in the groundwater underneath. The SI of different minerals from the PHREEQC model suggested that the majority of groundwater was either supersaturated or near saturation in terms of anhydrite, gypsum, and iron oxide mineral phases, including goethite (FeOOH) and hematite (Fe_2_O_3_), which precipitated As in groundwater. In contrast, the negative SI of aragonite, calcite (CaCO_3_^−^), dolomite ((CaMg(CO_3_)_2_)), and halite (NaCl) explained the evolution and mobilization of As in groundwater, and As release and mobility was associated with the dissolution of calcium carbonate, bicarbonate, and salt minerals [70,71]. Additionally, the fewer amounts of minerals such as FeCO_3_ react with natural calcium carbonate minerals, possibly causing their dissolution in the aquifer and releasing As into groundwater [70,79]. The presence of natural organic compounds and microbial activity may also be the reason to contaminate the groundwater [80,81,82,83]. Anthropogenic sources, the dissolution of CaCO_3_^−^, and coal mining are probable sources of sulfur [70,84,85]. The positive correlations between As and pH in all three tehsils hinted at the critical roles of pH in As release in the study area [49], and the increase in the mineralization rates were also assumed to play a major role in the evolution mechanism of As [86].

**Figure 5:**
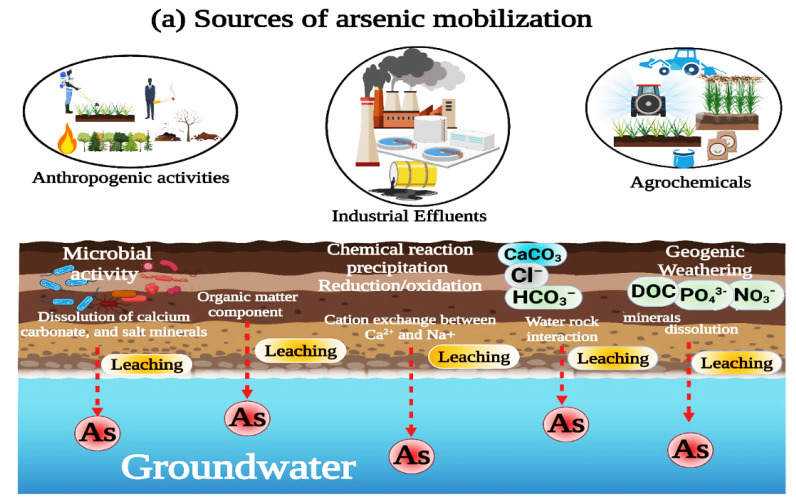

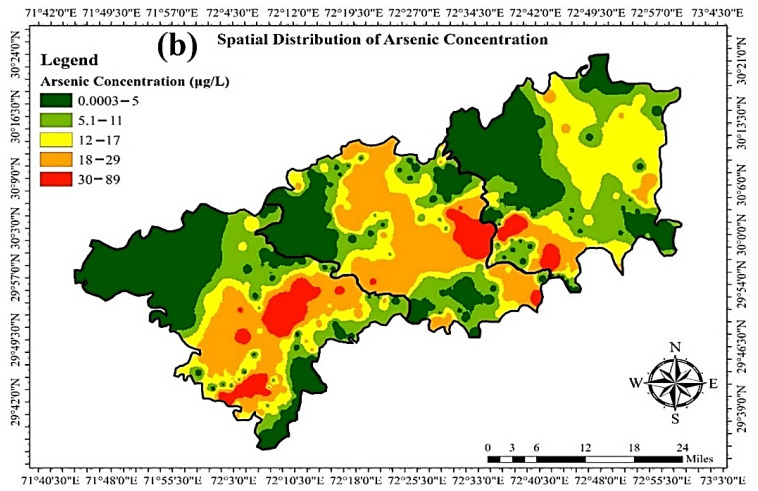
(**a**) Sources of As mobilization. (**b**) Spatial distribution of groundwater As in study area.

The groundwater contamination might be interrelated with the geochemical processes, as shown in Figure 5a. The spatial distribution of As was mapped through the inverse distance weight (IDW) interpolation (Figure 5b). The fluctuating As concentrations in groundwater was attributed to different reasons, mainly due to the higher urbanization, industrialization, and coal mining in the Vehari and Mailsi tehsils compared to Burewala. It can also be clearly seen that the river crosses almost through middle of the Vehari and Mailsi; therefore, there are higher As concentrations in the Vehari and Mailsi study area than in Burwala. Baloch et al. (2021) reported an average As concentration of 1.53 μg/L in Sakrand Sindh, which is lower than in this study and others reported high concentrations in Punjab [73,87]. 

### 3.4. Health Risk Assessment

**Figure 6:**
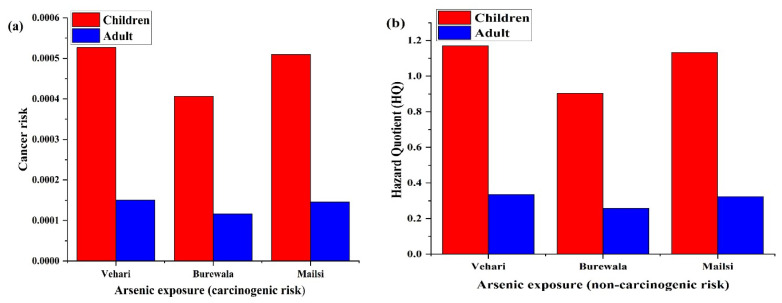
(**a**) Carcinogenic risk (CR) of adults and children. (**b**) Non-carcinogenic risk hazard quotient (HQ) of adults and children.

Health risks from groundwater As contamination were estimated by CDI, HQ, and CR for children and adults by considering drinking as the main exposure to As (Table S6). In this study, the CDI of groundwater in Vehari had an average value of 3.50 × 10^−4^ and 1.0 × 10^−4^ μg/(kg·day) for both children and adults, respectively. The average HQ was 1.17 and 0.30 for both children and adults, respectively, and the cancer risks were 5.27 × 10^−4^ and 1.51 × 10^−4^. For groundwater Burewala, the average CDI, HQ, and cancer risks were 2.71 × 10^−4^ (children) and 7.75 × 10^−5^ μg/(kg·day) (adults), 0.9 (children) and 0.25 (adults), and 4.07 × 10^−4^ (children) and 1.16 × 10^−4^ (adults), respectively. The groundwater in Mailsi exhibited slightly higher CDI (3.40 × 10^−4^ for children and 1.36 × 10^−4^ for adults), HQ (1.13 for children and 0.45 for adults), and cancer risk (5.10 × 10^−4^ for children and 2.04 × 10^−4^ for adults). Overall, the groundwater in Vehari and Mailsi exhibited more risk than that in Burewala. The carcinogenic risk was (CR > 10^−4^) in all three tehsils, indicating considerable cancer risks for adults and children (Figure 6a). There was significant non-carcinogenic risk for children (HQ > 1), which was higher than adults (Figure 6b). 

The CDI in this study was similar to those reported in Punjab, averaging 3.4 × 10^−4^ μg/(kg·day) [88], slightly higher than previous investigations in Khyber Pakhtunkhwa [0 to 5.6 × 10^−4^ μg/(kg·day)] [89] and Lahore [0.11–3.7 × 10^−4^ μg/(kg·day)] [90]. However, they were much lower than those reported in Bangladesh [50–500 × 10^−4^ μg/(kg·day)], which were caused by the elevated arsenic levels in Bangladesh’s groundwater [91], and Turkey [0.023–5.2 × 10^−4^ μg/(kg·day)] [92]. In this study, carcinogenic risk indices showed non-neglectable health threats to both children and adults regarding As exposure, while non-carcinogenic risks were only obvious for children in Vehari and Mailsi. Shakoor et al. (2016) also reported a high HQ (0.12–18.5) in Punjab [88]. These findings suggested that people in study area who ingest As-contaminated groundwater face significant cancer risks, such as stomach cancer, spontaneous abortions, diabetes, and gastric and esophageal complications. The As level in groundwater needs constant monitoring and remediation to protect the health of local people. 

## 4. Conclusions

In this study, we used statistical analyses and hydrogeochemical modeling to understand groundwater hydrogeochemistry, the evolution mechanism of As, and related health risks in Vehari, Burewala, and Mailsi (Punjab, Pakistan); levels were 14.0 μg/L, 11.0 μg/L, and 13.0 μg/L, respectively. Piper plots indicated that groundwater Na^+^-SO_4_^2−^, Ca^2+^, and Mg^2+^ types were more dominant and ionic ratios showed that the As contamination in groundwater was influenced by the dissolution of calcium carbonate. Furthermore, geochemical modeling showed negative saturation indices with calcium carbonate and salt minerals, including aragonite (CaCO_3_), calcite (CaCO_3_), dolomite [CaMg(CO_3_)_2_], and halite (NaCl). Findings suggested their critical roles in As mobilization/release in aquifers, also evidenced by multivariate analysis. The current study proposed the clear concept of the As evolution mechanisms in the aquifer, as evidenced by the inverse model, namely calcium-bearing minerals, chemical weathering, the exchange of cations between Ca^2+^ and Na^+^, and anthropogenic activities. Geomorphology and mineralogical geochemistry are important in order to understand how the dissolution of mineral phases is determined the As-mobilization in the groundwater and how the agrochemical utilization in agricultural lands should be properly treated to protect groundwater resources in Pakistan. The spatial distribution of As followed the order of Vehari > Mailsi > Burewala. The possible cancer risk (CR > 10^−4^) was significant for both children and adults, while the non-carcinogenic risk hazard quotient was only significant for children. It is strongly recommended that decision-makers in Pakistan should adopt immediate management actions and develop long-term strategies for protecting groundwater resources from As pollution.

## Figures and Tables

**Table 1:** Statistical physicochemical parameters of groundwater in three tehsils. All parameters are in (mg/L), except for pH, As (µg/L), EC (µS/cm), and turbidity (NTU). Below detection limit (BDL).

Parameters	Vehari (*n* = 170)		Burewala (*n* = 170)		Mailsi (*n* = 170)		WHO (2022) Standard
	Min–Max	Mean ± Standard	Min–Max	Mean ± Standard	Min–Max	Mean ± Standard	
EC	308–4550	1569.94 ± 848.63	85–4400	1230.78 ± 646.37	226–2690	1185.059 ± 468.99	1000
TDS	234–3148	1072.12 ± 514.8	277–3173	896.68 ± 429.54	359–3298	896.64 ± 385.68	1000
pH	6.85–7.61	7.17 ± 0.17	6.78–7.15	6.98 ± 0.07	7.2–8.35	7.66 ± 0.23	6.5–8.5
Turbidity	0.3–991	26.37 ± 86.34	0.3–202	15.98 ± 38.57	0.2–4.2	1.22 ± 1.12	4.0
Alkalinity	2.2–610	10.82 ± 46.56	2.8–13.2	5.98 ± 1.81	1.4–12.8	6.50 ± 1.68	-
Hardness	100–820	368.47 ± 132.94	105–820	348.85 ± 123.71	190–1540	414.58 ± 152.34	-
HCO_3_^−^	110–900	345.08 ± 116.35	80–660	299.20 ± 92.78	70–640	324.23 ± 84.59	-
Cl^−^	10–518	107.94 ± 88.83	10–502	74.08 ± 57.8	28–336	103.51 ± 53.84	200–300
SO_4_^2–^	41–1300	316.87 ± 219.81	18–1432	244.24 ± 185.36	18–840	167.49 ± 126.37	250
Ca^2+^	24–192	86.31 ± 35.40	26–208	86.44 ± 32.59	8–320	86.91 ± 40.77	200
Mg^2+^	6–98	36.65 ± 16.55	10–95	32.25 ± 14.52	2–180	48 ± 21.55	150
Na^+^	14–850	195.29 ± 157.17	13–620	131.78 ± 109.08	21–360	107.97 ± 68.98	200
K^+^	2.6–42.6	9.53 ± 6.53	3.7–69	8.085 ± 6.24	1–74	9.49 ± 8.29	12
NO_3_^−^	BDL-17.66	1.51 ± 3.07	0.01–15.82	1.8 ± 3.31	BDL-14	1.85 ± 2.94	50
F^−^	BDL-3.15	0.59 ± 0.37	0.18–1.35	0.46 ± 0.20	0.22–1.24	0.51 ± 0.17	1.5
Fe^2+^	0.01–2.94	0.18 ± 0.39	0.01–3.92	0.19 ± 0.43	0.01–2.94	0.23 ± 0.40	0.3
As	BDL-45	14.0 ± 11.85	BDL-52	11.0 ± 11.62	BDL-89	13.0 ± 15.3	10

**Table 2:** Factor loading for groundwater physicochemical parameters in the study area.

Tehsil	Vehari			Burewala			Mailsi		
Component	PC1	PC2	PC3	PC1	PC2	PC3	PC1	PC2	PC3
EC	**0.964**	−0.199	0.092	**0.957**	−0.094	−0.185	**0.869**	0.063	0.195
TDS	**0.981**	0.051	0.131	**0.962**	0.141	−0.16	**0.972**	−0.074	0.027
pH	0.069	−0.062	0.376	−0.037	0.125	−0.14	−0.34	0.208	**0.512**
Turbidity	0.017	0.245	0.329	−0.052	0.352	0.016	0.217	**0.745**	0.086
Alkalinity	0.072	−0.202	0.014	**0.837**	−0.215	0.176	**0.814**	0.191	−0.222
HCO_3_^−^	**0.707**	−0.067	−0.108	**0.821**	−0.254	0.182	**0.823**	0.178	−0.209
Cl^−^	**0.867**	−0.207	0.097	**0.844**	−0.057	−0.114	**0.868**	−0.048	0.132
SO_4_^2–^	**0.936**	−0.111	0.193	**0.856**	0.038	−0.356	**0.862**	−0.081	0.28
Ca^2+^	0.392	**0.831**	0.157	**0.592**	0.706	0.173	**0.779**	−0.334	−0.025
Mg^2+^	**0.816**	0.301	−0.155	**0.833**	0.128	0.193	**0.617**	0.206	−0.502
Na^+^	**0.856**	−0.433	0.121	**0.859**	−0.307	−0.332	**0.792**	0.13	0.383
K^+^	0.331	0.108	−0.556	0.301	−0.124	0.481	**0.573**	−0.097	0.291
Hardness	**0.662**	0.71	0.026	**0.793**	0.527	0.208	**0.88**	−0.104	−0.307
NO_3_^−^	0.125	−0.064	−0.637	0.328	−0.006	**0.648**	0.322	−0.502	0.062
Fe	0.154	0.093	−0.072	0.156	−0.12	0.039	−0.04	0.307	−0.002
F^−^	0.466	−0.643	−0.153	**0.517**	−0.459	−0.148	0.225	0.303	0.313
As	−0.288	−0.257	**0.514**	−0.002	**0.663**	−0.378	0.025	**0.783**	−0.12
Eigen Value	6.46	2.18	1.40	7.56	1.85	1.32	7.56	1.92	1.19
% of Variance	38.04	12.83	8.27	44.49	10.88	7.77	44.51	11.33	7.01
Cumulative %	38.04	50.88	59.15	44.49	55.37	63.15	44.51	55.85	62.86

Extraction method: principal component analysis. Rotation method: Kaiser normalization of Varimax. Bold values show higher loading value.

## Data Availability

The data presented in this study are available on request from the first or corresponding authors.

## Supplementary Materials

The following supporting information can be downloaded at: https://www.mdpi.com/article/10.3390/ijerph192013325/s1.
