# Supplementary material for: Evolution Mechanism of Arsenic Enrichment in Groundwater and Associated Health Risks in Southern Punjab, Pakistan
Source: Int J Environ Res Public Health. 2022 Oct 15;19(20):13325. doi: 10.3390/ijerph192013325 (PMC9603767; doi:10.3390/ijerph192013325)
Supplement: Supplementary file 1 [file ijerph-19-13325-s001.zip › ijerph-1901439-supplementary.pdf]

Supplementary File

Evolution Mechanism of Arsenic Enrichment in Groundwater and Associated Health Risks in Southern Punjab, Pakistan

Muhammad Yousuf Jat Baloch [1,2], Wenjing Zhang [1,2,*], Dayi Zhang [1,2], Baig Abdullah Al Shoumik [3], Javed Iqbal [4], Shuxin Li [1,2], Juanfen Chai [1,2], Muhammad Ansar Farooq [5] and Anand Parkash [6]

1 Key Laboratory of Groundwater Resources and Environment, Ministry of Education, Jilin University, Changchun 130021, China
2 College of New Energy and Environment, Jilin University, Changchun 130021, China
3 Soil, Water and Environment Discipline, Khulna University, Khulna 9208, Bangladesh
4 School of Environmental Studies, China University of Geosciences, Wuhan 430074, China
5 Institute of Environmental Sciences and Engineering, School of Civil and Environmental Engineering, National University of Science and Technology, Islamabad 44000, Pakistan
6 School of Chemistry and Chemical Engineering, Shaanxi Normal University, Chang'an West Street 620, Xi'an 710119, China
* Correspondence: zhangwenjing80@hotmail.com

Table. S1. Saturation index values of various minerals phases in the study area.

Mineral compositions	Vehari				Burewala				Mailsi			
	Mean	Minimum	Maximum	SD	Mean	Minimum	Maximum	SD	Mean	Minimum	Maximum	SD
Anhydrite ($CaSO_4$)	0.71	0.05	1.30	0.26	0.68	-0.13	1.35	0.25	0.54	-0.53	1.48	0.33
Aragonite ($CaCO_3$)	-2.01	-2.61	3.55	0.50	-2.04	-2.63	-1.45	0.18	-1.96	-2.82	-1.59	0.18
Calcite $CaCO_3$)	-1.87	-2.47	3.6	0.50	-1.89	-2.49	-1.31	0.18	-1.82	-2.68	-1.44	0.18
Dolomite [$CaMg(CO_3)_2$]	-4.04	-5.16	7.24	0.98	-4.15	-5.33	-3.21	0.35	-3.80	-5.44	-3.12	0.33
Goethite (FeOOH)	0.160	-1.24	9.64	0.90	0.24	-0.96	1.80	0.52	0.39	-1.64	1.85	0.48
Gypsum ($CaSO_4 \cdot 2H_2O$)	1.00	0.35	1.57	0.25	0.97	0.16	1.61	0.24	0.83	-0.23	1.76	0.33
Halite (NaCl)	-3.90	-5.67	-2.57	0.59	-4.18	-5.70	-2.61	0.56	-4.01	-5.07	-2.96	0.42
Hematite (Fe_2O_3)	2.33	-0.45	21.30	1.81	2.50	0.09	5.62	1.04	2.80	-1.27	5.719	0.97

Table. S2. Pearson's correlation matrix of physiochemical parameters in Vehari (*n* = 170).

	EC	TDS	pH	Turbidity	Alkalinity	HCO_3^-	Cl^-	SO_4^{2-}	Ca^{2+}	Mg^{2+}	Na^+	K^+	Hardness	NO_3^-	Fe^{2+}	F^-	As
**EC**	1																
**TDS**	.952**	1															
**pH**	0.08	0.091	1														
**Turbidity**	-0.007	0.048	0.068	1													
**Alkalinity**	0.068	0.054	-0.022	-0.021	1												
**HCO_3^-**	.679**	.633**	-0.118	-0.013	.165*	1											
**Cl^-**	.905**	.854**	0.148	-0.044	0.039	.462**	1										
**SO_4^{2-}**	.949**	.968**	0.123	0.041	0.071	.553**	.828**	1									
**Ca^{2+}**	.222**	.464**	-0.029	.176*	-0.058	.223**	.173*	.312**	1								
**Mg^{2+}**	.690**	.774**	0.043	-0.013	-0.032	.529**	.613**	.674**	.446**	1							
**Na^+**	.934**	.841**	0.072	-0.025	0.126	.673**	.831**	.885**	-0.001	.485**	1						
**K^+**	.253**	.249**	-0.03	0.014	-0.027	.266**	.228**	.197*	0.078	.348**	.165*	1					
**Hardness**	.485**	.687**	-0.001	0.105	-0.057	.409**	.415**	.536**	.877**	.793**	.236**	.222**	1				
**NO_3^-**	0.071	0.068	-0.046	-0.098	-0.033	0.069	0.119	0.022	-0.034	0.15	0.078	.224**	0.049	1			
**Fe^{2+}**	0.111	0.126	-0.035	-0.055	-0.015	0.006	0.143	0.108	0.063	.191*	0.054	-0.004	0.137	-0.061	1		
**F^-**	.524**	.388**	-0.01	-0.076	0.086	.323**	.446**	.468**	-.328**	.263**	.574**	0.103	-0.096	0.143	0.077	1	
**As**	-.180*	-.210**	0.026	-0.005	-0.051	-.191*	-0.136	-.155*	-.183*	-.347**	-0.084	-.259**	-.293**	-0.142	-.151*	-0.103	1

*correlation is significant at p < 0.05, **correlation is significant at p < 0.01, n means number of samples

Table. S3. Pearson's correlation matrix of physiochemical parameters in Burewala (*n* = 170).

	EC	TDS	pH	Turbidity	Alkalinity	HCO_3^-	Cl^-	SO_4^{2-}	Ca^{2+}	Mg^{2+}	Na^+	K^+	Hardness	NO_3^-	Fe^{2+}	F^-	As
**EC**	1																
**TDS**	.946**	1															
**pH**	-0.024	-0.013	1														
**Turbidity**	-0.064	-0.003	-0.022	1													
**Alkalinity**	.755**	.688**	-0.04	-0.09	1												
**HCO_3^-**	.740**	.667**	-0.049	-0.126	.972**	1											
**Cl^-**	.864**	.805**	-0.021	-0.071	.655**	.646**	1										
**SO_4^{2-}**	.901**	.943**	-0.006	-0.009	.540**	.522**	.676**	1									
**Ca^{2+}**	.458**	.645**	0.046	0.134	.382**	.346**	.429**	.461**	1								
**Mg^{2+}**	.717**	.789**	-0.06	-0.012	.665**	.651**	.637**	.649**	.521**	1							
**Na^+**	.936**	.850**	-0.038	-0.098	.717**	.711**	.805**	.869**	.210**	.562**	1						
**K^+**	.226**	.213**	-0.038	-0.063	.285**	.273**	.219**	0.136	0.124	.311**	0.137	1					
**Hardness**	.648**	.807**	0.001	0.08	.574**	.544**	.591**	.618**	.910**	.828**	.410**	.233**	1				
**NO_3^-**	.249**	.235**	-0.039	-0.031	.287**	.293**	.217**	0.125	.269**	.301**	0.141	.163*	.323**	1			
**Fe^{2+}**	0.108	0.117	-0.014	-0.068	.233**	.216**	0.066	0.093	0.083	0.061	.154*	-0.027	0.085	0.012	1		
**F^-**	.531**	.445**	-0.055	-0.085	.430**	.430**	.364**	.485**	0.001	.362**	.546**	.198**	.175*	0.146	0.006	1	
**As**	-0.007	0.097	0.034	0.032	-0.126	-0.15	-0.029	0.089	.336**	-0.032	-0.083	-0.104	.206**	-.184*	-0.063	-.162*	1

*correlation is significant at $p < 0.05$, **correlation is significant at $p < 0.01$, n means number of samples

Table. S4. Pearson's correlation matrix of physiochemical parameters in Mailsi ($n = 170$).

	EC	TDS	pH	Turbidity	Alkalinity	HCO_3^-	Cl^-	SO_4^{2-}	Ca^{2+}	Mg^{2+}	Na^+	K^+	Hardness	NO_3^-	Fe^{2+}	F^-	As
**EC**	1																
**TDS**	.802**	1															
**pH**	-.26**	-.292**	1														
**Turbidity**	.233**	.162*	0.087	1													
**Alkalinity**	.670**	.702**	-.260**	.218**	1												
**HCO_3^-**	.679**	.713**	-.258**	.212**	.980**	1											
**Cl^-**	.831**	.859**	-.338**	.152*	.571**	.571**	1										
**SO_4^{2-}**	.736**	.925**	-0.15	0.117	.538**	.546**	.759**	1									
**Ca^{2+}**	.592**	.822**	-.350**	-0.001	.522**	.543**	.660**	.699**	1								
**Mg^{2+}**	.411**	.611**	-.202**	.256**	.556**	.555**	.462**	.419**	.277**	1							
**Na^+**	.811**	.750**	-0.143	.196*	.661**	.668**	.789**	.796**	.453**	.233**	1						
**K^+**	.481**	.545**	-0.097	0.079	.383**	.396**	.416**	.569**	.412**	.257**	.424**	1					
**Hardness**	.635**	.906**	-.351**	0.149	.672**	.685**	.711**	.713**	.829**	.767**	.441**	.426**	1				
**NO_3^-**	.268**	.295**	-0.04	-.166*	.194*	.207**	.226**	.212**	.362**	.165*	0.119	.206**	.339**	1			
**Fe^{2+}**	-0.038	-0.038	0.042	.189*	-0.006	-0.015	-0.063	-0.014	-0.061	0.001	-0.035	-0.007	-0.04	-0.076	1		
**F^-**	.210**	.177*	0.006	.313**	0.132	0.149	.194*	0.14	0.124	0.118	.177*	0.101	.151*	0.108	-0.01	1	
**As**	0.07	-0.023	0.068	.403**	0.147	0.138	-0.018	-0.04	-.189*	.160*	0.094	-0.06	-0.032	-.328**	0.1	0.118	1

*correlation is significant at $p < 0.05$, **correlation is significant at $p < 0.01$, n means number of samples

Table. S5. Physicochemical composition of six groundwater samples in mg/L, except As (μg/L) and pH used in the inverse modeling and results of mass transfer calculation along flow-path.

Parameters	Path I			Path II			Mineral phases	Process	Path I		Path II	
	A	B	C	X	Y	Z			$A \rightarrow B$	$B \rightarrow C$	$X \rightarrow Y$	$Y \rightarrow Z$
**pH**	6.93	7.42	7.45	7.2	7.19	7.52	Anhydrite ($CaSO_4$)		-0.0277	-6.539	-1.97	–
**TDS**	614	862	722	654	938	1274	Aragonite ($CaCO_3$)		-0.01162	-0.3343	0.01496	0.02299
**Na^+**	38	93	97	67	97	160	Calcite ($CaCO_3$)	Mineral dissolution or precipitation	-0.01491	0.01113	0.00005814	-0.01724
**Ca^{2+}**	80	100	88	92	110	152	Dolomite [$CaMg(CO_3)_2$		-0.00204	0.006132	0.02315	0.005807
**Mg^{2+}**	24	39	36	22	28	49	Goethite (FeOOH)		0.00003703	-0.00001053	0.000002681	0.00002438
**HCO_3^-**	225	350	360	270	250	400	Gypsum ($CaSO_4$:$2H_2O$)		–	1.517	–	0.0707
**Cl^-**	34	48	84	53	62	182	Halite (NaCl)		0.008068	0.04089	0.05767	0.01149
**K^+**	4.7	6.3	8	6.2	5.1	9	Hematite Fe_2O_3		0.00001851	-0.000005263	0.000001341	0.00001254
**Fe^{2+}**	0.09	0.3	0.12	0.14	0.07	0.23	H_2O (g)	Evaporation	-2.94	1.03	3.93	-1.5
**NO_3^-**	0.15	0.3	0.47	2.71	0.32	1.12	CO_2(g)		-0.01689	0.07148	0.1465	-0.3339
**SO_4^{2-}**	146	185	100	115	270	210	CaX_2	Cation Exchange	-0.01562	0.007318	0.01642	0.00596
**As**	14	27	50	11	21	30	NaX		0.03124	-0.01464	0.03283	-0.01192

Negative values mean phase removal from groundwater, while positive means phase dissolution. All mass transfers values in mmol/L

Table. S6. Chronic daily intake (CDI), Non carcinogenic risk hazard quotient (HQ), and carcinogenic risk (CR) of As exposure by adult and children in the study area.

	Vehari						Burewala						Mailsi					
	**CDI**		**Hazard Quotient**		**CR**		**CDI**		**Hazard Quotient**		**CR**		**CDI**		**Hazard Quotient**		**CR**	
	Children	Adult	Children	Adult	Children	Adult	Children	Adult	Children	Adult	Children	Adult	Children	Adult	Children	Adult	Children	Adult
Mean	3.50×10^{-4}	$1.0E\times10^{-4}$	1.17	0.30	5.27×10^{-4}	1.51×10^{-4}	2.71×10^{-4}	7.7×10^{-5}	0.9	0.25	4.07×10^{-4}	1.16×10^{-4}	3.40×10^{-4}	1.36×10^{-4}	1.13	0.45	5.10×10^{-4}	2.04×10^{-4}
Minimum	0	0	0	0	0	0	0	0	0	0	0	0	0	0	0	0	0	0

Maximum	1.13×10^{-3}	3.21×10^{-4}	3.75	1.07	1.69×10^{-3}	4.82×10^{-4}	1.30×10^{-3}	3.71×10^{-4}	4.33	1.24	1.95×10^{-3}	5.57×10^{-4}	2.23×10^{-3}	8.90×10^{-4}	7.42	2.97	3.34×10^{-3}	1.34×10^{-3}
S.D	2.96×10^{-4}	8.47×10^{-5}	9.88×10^{-1}	2.82×10^{-1}	4.45×10^{-4}	1.27×10^{-4}	2.91×10^{-4}	8.31×10^{-5}	9.69×10^{-1}	2.77×10^{-1}	4.36×10^{-4}	1.25×10^{-4}	3.85×10^{-4}	1.54×10^{-4}	1.28	0. 51	5.77×10^{-4}	2.31×10^{-4}
